# Beyond the healthy immigrant paradox: decomposing differences in birthweight among immigrants in Spain

**DOI:** 10.1186/s12992-020-00612-0

**Published:** 2020-09-24

**Authors:** Mikolaj Stanek, Miguel Requena, Alberto del Rey, Jesús García-Gómez

**Affiliations:** 1 Department of Sociology and Communication, University of Salamanca, Edificio F.E.S. Avda Francisco Tomás y Valiente s/n Salamanca, 37007 Salamanca, Spain; 2 Department of Sociology II, UNED, C/Obispo Trejo 2, 28040 Madrid, Spain

**Keywords:** Spain; healthy immigrant paradox, Perinatal health, Compositional heterogeneity

## Abstract

**Background:**

The healthy immigrant paradox refers to the unexpected health advantages of immigrant groups settled in host countries. In this population-based study we analyze immigrant advantages in birthweight decomposing differences between infants born to immigrant mothers from specific origins.

**Method:**

Using publicly available data from Spanish Vital Statistics for the period 2007–2017, differential birthweights among several groups of immigrants were estimated with an ordinary least squares regression. The Oaxaca–Blinder regression-based decomposition method was then applied to identify the extent to which differences in birthweight between groups corresponded to compositional disparities or to other factors.

**Results:**

Our analysis of singleton live births to migrant mothers in Spain between 2007 and 2017 (*N* = 542,137) confirmed the healthy immigrant paradox for certain immigrant populations settled in Spain. Compared with infants born to mothers from high-income countries, the adjusted birthweight was higher for infants born to mothers from non-high- income European countries (33.2 g, 95% CI: 28.3–38.1, *P* < 0.01), mothers from African countries (52.2 g, 95% CI: 46.9–57.5, *P* < 0.01), and mothers from Latin American countries (57.4 g, 95% CI: 52.9–61.3, *P* < 0.01), but lower for infants born to mothers from Asian non-high-income countries (− 31.4 g, 95% CI: − 38.4 to − 24.3, *P* < 0.01). Decomposition analysis showed that when compared with infants born to mothers from high-income countries, compositional heterogeneity accounts for a substantial proportion of the difference in birthweights. For example, it accounts for 53.5% (95% CI: 24.0–29.7, *P* < 0.01) of the difference in birthweights for infants born to mothers from non-high-income European countries, 70.9% (95% CI: 60–66.7, *P* < 0.01) for those born to mothers from African countries, and 38.5% (95% CI: 26.1–29.3, *P* < 0.01) for those born to mothers from Latin American countries.

**Conclusions:**

Our results provide strong population-based evidence for the healthy immigrant paradox in birthweight among certain migrant groups in Spain. However, birth outcomes vary significantly depending on the origins of migrant subpopulations, meaning that not all immigrant groups are unexpectedly healthier. A significant portion of the perinatal health advantage of certain immigrant groups is only a by-product of their group composition (by age, parity, marital status, socioeconomic status, and citizenship of mother, age and migratory status of father and type of delivery) and does not necessarily correspond to other medical, environmental, or behavioral factors.

## Background

The healthy immigrant paradox describes a phenomenon observed in many countries where immigrants who have just moved to a host country have better health outcomes than the native populations of the host country. An interesting variation of this paradox is when immigrants from high-income societies have worse health results in the host countries than immigrants from low- and middle-income societies. These phenomena have been rightly considered “paradoxical” as migrants coming from the developing world are more exposed to adverse conditions in host countries, which would be expected to take an immediate toll on their health [1, 2]. Perinatal health provides a good case in point for these puzzling outcomes. Associations between birthweight and maternal ethnic origin among migrant women have been documented in diverse contexts [3–5]. There is evidence of better health outcomes among immigrant children when compared with native children in terms of low birthweight, infant mortality rate and premature births, despite more difficult life conditions [6, 7]. Nevertheless, research approaches including country-specific analyses [8–10], systematic cross-country analyses [11, 12], and meta-analytical reviews [13, 14] have yielded divergent findings and provided conflicting conclusions. The results may vary depending on both substantive and methodological elements. The host country, maternal origin, and specific health outcomes (birthweight, mortality and premature births) often involve different observations, conceptual definitions, and research designs, and all of these measurement problems may further complicate the findings. As Gagnon et al. [13] suggested, efforts to ensure research clarity and homogeneity are needed to confirm and properly understand these associations.

To date, several explanations for the healthy immigrant paradox have been provided. One of the most popular explanations is the immigrant selectivity hypothesis. This hypothesis is based on the assumption that immigrants are not a random sample of their home country populations. Those who choose to migrate may differ from their home country population, and this selection may occur on many observable and unobservable traits, including socio-economic characteristics and health [15]. In contrast, the ethnic maintenance hypothesis suggests that certain immigrant groups may exhibit specific socio-cultural traits (traditions, behaviors, and norms) that make positive perinatal outcomes more probable. More specifically, some migrant groups exhibit protective health behaviors based on internal social norms and social ties that may increase birthweight even under adverse socio-economic conditions. These behaviors may including healthier diets, less consumption of tobacco and alcohol, and increased social support from family members and their ethnic communities [16, 17].

Empirical findings from a vast body of research have revealed several social factors that have a significant impact on immigrant birthweight. There is evidence that the socio-economic status, in terms of educational attainment and occupational position, has a positive impact on the health of mothers and their children through improved work and economic conditions, psycho-social resources, and healthy lifestyle choices [18–20]. However, this relationship can be curvilinear, with university-educated mothers presenting adverse birth outcomes likely due to their older age at delivery [6]. Several studies have also provided evidence of an association between the marital status and birth outcomes. Single mothers face worse economic circumstances, experience greater psychological stress during pregnancy, and are less likely to seek timely prenatal care—all factors that increase the risk of low birthweight [21]. The available evidence concerning the effects of time of residence in the host country and level of naturalization is ambiguous. Teitler et al. [22] showed that there was a systematic reduction in average birthweight during the first decade after arrival to the United States although the effect size relies strongly on the country of origin. In contrast, Juarez and Hjern [23] did not find a similar pattern among immigrants in Sweden. Sow et al. [12] showed that adopting Belgian nationality has a favorable effect on birth outcomes including birthweight. More generally, Sørbye et al. [24] found that the birthweight of immigrants does not correlate with integration policies in the host country.

Nulliparity is also usually associated with a significantly increased risk of low birthweight. However, grand multiparity and great grand multiparity are also associated with low birthweight [25]. Non-linear patterns have been observed for the impact of maternal age at birth on birthweight. More specifically, several studies have shown a U-shaped relationship between maternal age and birthweight, with infants born to the youngest (younger than 15 years) and the oldest (40 years and older) mothers more at risk of low birthweights [26, 27].

Spain is a very good case in point to analyze the health outcomes of immigrants. In the last two decades, Spain has become a receiving country for immigrants from Latin America, Africa, Asia and high- and non-high-income European countries [28] and its immigrant population has grown from 1.6% in 1998 to 14.3% in 2019. Massive inflows of woman in reproductive ages provide an excellent opportunity to analyze the reproductive and perinatal health outcomes of migrant subpopulations in high-income countries [29–31].

Available data from Spain seem to confirm the healthy immigrant pattern in terms of perinatal health. The estimation of differences between the birthweights of native- and immigrant-born infants shows that the latter tend to weigh more [21, 32–34]. Fuster et al. [35] confirmed that this positive immigrant–native gradient has been consistently maintained from 1980 to 2010.

These theoretical and empirical studies are useful for understanding how and why the gradient in birthweights between immigrants from different origins depends on the specific composition of immigrant subpopulations. This includes the differential distributions of certain characteristics of mothers (age, parity, marital status, socioeconomic status and citizenship), fathers (age and migratory status), and births (with complications, caesarean), and their differential associations with birthweight across these migrant groups. On the one hand, younger, primiparous, non-married, less educated and low-class migrant mothers are expected to deliver babies with less weight. On the other, mothers without Spanish citizenship as well as older and migrant fathers tend to have heavier babies. Finally, complicated and cesarean deliveries should be positively associated to birthweight. Since these characteristics are not necessarily evenly distributed among different immigrant communities, stating specific predictions about their net aggregate impact on birthweight is not sensible until the respective composition of each group is known. However, considering these factors, we hypothesized that birthweight vary, to some extent, based on the distributions of specific features between groups of immigrants from different origins.

This study specifically aimed to decompose differences in birthweight between infants born to immigrant mothers from specific origins and to estimate how those differences are due to uneven distributions of relevant characteristics and/or other unspecified factors. First, we compared the composition of several immigrant groups to identify traits that might cause differences in birthweight. Second, we ran an ordinary least squares (OLS) linear regression model, with birthweight as the main outcome and the region of origin as the main exposure. The model’s results allowed us to estimate adjusted differences in birthweight between infants whose mothers have migrated from different origins. Lastly, using decomposition, we analyzed the extent to which differences in birthweight are associated with disparities in the composition of known characteristics of immigrant groups from different origins.

## Methods

To date, research on patterns and determinants of birthweight among immigrants in Spain has been limited by a relative scarcity of data. Researchers have usually used data from Spanish Vital Statistics (Estadistica del Movimiento Natural de la Población), which offers basic information regarding some characteristics of births, new-born infants, and their parents. Using this same data source and following previous population-based studies on birthweights in Spain [35–37], we did not restrict the analysis to a single calendar year, but extended our data to cover a 11-year period to obtain a higher number of immigrant mothers from different origins. Our research took advantage of aggregate data from several years to build a dataset reflecting the full number of births that occurred in Spain over the period 2007–2017. Our database provides an opportunity to analyze in greater depth several factors that are known to be associated with differences in birthweight between immigrant populations.

The original annual datasets are available at the Spanish National Statistical Office (Instituto Nacional de Estadística, INE) webpage and are fully accessible to the public. We have aggregated yearly individual microdata of all births registered in Spain from 2007 to 2017 into a new database. These registers include all births occurred in Spain irrespective of the legal status of the mother (e.g. documented, undocumented, refugee, student, temporary worker, etc.) and contain several characteristics of the deliveries and infants, as well as some sociodemographic information about their parents. It is important to emphasize that this new database constitutes a complete representation of all births that occurred in the country in the period considered. Although not strictly necessary, we also offer *p* values and confidence intervals as per convention, as plausible measures of the variability of our estimates.^1^

The main outcome variable was the registered birthweight, from the Spanish vital statistics data, for the period 2017–2017. The mean birthweight of infants born to immigrant mothers from different origins is a valid indicator of perinatal health, as well as a strong predictor of mid- and long-term health-related outcomes as has been shown in previous research [13, 24, 38]. It should be noted that there is a large correlation (point-biserial correlation coefficient: − 0.58, 95% CI: − 0.58 to − 0.58) between the mean birthweight at term and low birthweight (< 2500 g). For analytical purposes, we selected living singleton infants who were delivered in Spain from mothers with a foreign place of origin. To avoid undue heterogeneity that may be produced by potential outliers in terms of weights and gestational ages, we only selected births at term with credible weights, i.e., birthweights within the range of 250–5999 g and infants born at 37–41 weeks of gestation. After excluding cases with missing information in the relevant variables, 542,137 birth records were included in this analysis (Table 1).

**Table 1:** Characteristics of the study population by origin

	Native Spanish	High-income countries	Non-high-income European countries	Africa	Latin America	Asia	All migrant born
Singleton births	2,401,964	65,220	100,773	127,040	223,162	25,942	542,137
%	82%	12%	19%	23%	41%	5%	100.0%
Mean Birthweight	3281.7	3318.1	3368.3	3407.4	3390.1	3320.7	3378.1
Infant sex %							
Male	51.3	51.0	51.5	51.7	51.2	52.0	51.4
Female	48.7	49.0	48.5	48.3	48.8	48.0	48.6
Birth order %							
1st	54.4	53.9	58.0	38.4	49.0	44.4	48.5
2nd - 3th	44.1	44.0	40.1	52.0	47.1	51.0	46.8
4th+	1.5	2.1	1.9	9.6	3.9	4.5	4.7
Labor complications %							
Yes	13.1	13.9	12.2	14.2	15.9	11.6	14.4
No	86.9	86.1	87.8	85.8	84.1	88.4	85.6
Caesarean delivery %							
Yes	22.9	22.5	18.4	20.2	24.8	20.7	22.1
No	77.1	77.6	81.6	79.8	75.2	79.3	77.9
Age of mother %							
< 20	1.5	1.2	3.9	3.1	4.1	1.3	3.4
20–30	30.2	28.3	58.8	56.1	45.1	63.2	49.1
31+	68.3	70.6	37.3	40.8	50.8	35.5	47.6
Marital status of mother %							
Married/Partner	62.7	52.7	55.0	83.3	45.9	70.0	58.3
Never married	33.7	42.8	39.4	14.8	48.3	28.2	37.2
Separated/Divorced	3.4	4.2	5.3	1.8	5.6	1.7	4.3
Widow	0.2	0.2	0.2	0.2	0.2	0.1	0.2
Education of mother %							
Primary or less	8.4	7.5	18.0	61.1	14.9	41.1	26.6
Lower Secondary	20.4	17.7	33.8	20.9	32.4	31.1	28.1
Upper Secondary	30.1	30.6	30.5	12.5	33.6	18.5	27.0
University	41.1	44.3	17.8	5.5	19.2	9.3	18.3
Family class %							
Manager/Professional	16.2	19.6	5.6	1.8	7.9	3.9	7.2
White Collar	26.0	27.4	9.4	3.3	12.2	4.6	11.0
Service Workers	17.3	18.4	15.8	8.6	20.1	41.3	17.4
Skilled Workers	15.4	9.4	13.1	8.7	7.8	3.6	9.0
Unskilled Workers	7.4	6.6	19.6	15.1	11.5	9.6	13.2
Inactive	17.7	18.7	36.6	62.6	40.6	37.0	42.2
Citizenship of mother %							
Spanish	99.8	38.9	1.5	6.8	31.8	5.2	19.9
Other	0.2	61.1	98.5	93.2	68.2	94.9	80.1
Mean Age of father	34.4	35.3	33.0	36.8	33.5	32.5	34.3
Migratory status of father							
Native	94.3	64.3	24.7	7.7	34.6	6.9	28.7
Migrant	5.7	35.7	75.4	92.3	65.4	93.2	71.3

Our main exposure variable was the mother’s country of origin, which was coded into the following five main groups of countries according to their development level and geographic location: high-income countries (HICs: Austria, Belgium, Denmark, Finland, France, Germany, Greece, Ireland, Italy, Luxembourg, Netherlands, Portugal, Spain, Sweden, Switzerland, Monaco, Andorra, Norway, United Kingdom, Israel, United States, Canada, Australia, New Zealand, Japan, South Korea, Taiwan, and Singapore); non-high-income European countries (NHI-EUR: Albania, Armenia, Belarus, Bosnia, Bulgaria, Croatia, Cyprus, Czech Republic, Estonia, Georgia, Hungary, Latvia, Lithuania, Macedonia, Malta, Moldavia, Poland, Romania, Serbia, Slovakia, Slovenia, and Ukraine); Latin American countries (LACs); African countries (AFCs); and Asian countries (ASCs) not previously included in HICs. The distinction between high-income and non-high-income countries has been established following the 2007 World Bank’s classification according to which the threshold for high-income is a gross national income (GNI) per capita of 11,455 US dollars [39].

In our analysis, we included several covariates to control for possible confounders. Birth characteristics included infant sex (male, female), birth order (1st, 2nd–3rd, and 4th+), labor complications (yes, no) and cesarean delivery (yes, no). The mother’s characteristics included age at birth (< 20, 20–30, and 31+ years), marital status (married/in consensual union, never married, separated/divorced, and widow), and citizenship (Spanish, other). As a measure of socio-economic status (SES), we used the mother’s educational attainment (primary or less, lower secondary, upper secondary, and university). To complement the measure of SES, we included the highest occupational status of the mother or father as a measure of family occupational class (manager/professional, white collar, service workers, skilled workers, unskilled workers, and inactive). Two of the father’s characteristics—age and migratory status (native [born in Spain], migrant [born abroad])—were also included. Table 1 reports the main characteristics of the study population.

We used two analytical approaches. First, we applied OLS regression models to estimate the effects of the different exposures, in particular the country of origin and the socio-demographic and socio-economic variables, on birthweight. Regression modelling implicitly assumes that the distribution of features is similar for all individuals in the sample. Therefore, if there are substantial differences in the distributions of certain features, regression might be insufficient to capture their effects on the study outcomes. To overcome this limitation we applied a regression-based decomposition technique, developed by Blinder [40] and Oaxaca [41], to differences in birthweights observed between several immigrant groups. This technique allows any difference to be decomposed into two portions: (1) the “composition effect” or “explained component” that corresponds to the varying distributions of traits of the groups being compared; (2) the “coefficient effect” or “unexplained component” that corresponds to the other factors associated with birthweight that are specific to these groups. The explained component accounts for the part of the difference that is attributable to structural or compositional dissimilarities between the groups, whereas the unexplained component accounts for the behavioral differences between the groups that are not attributable to their composition. The composition component indicates differences in birthweight that would be observed between the groups if the regression coefficients of independent variables on birthweight in each group had been the same while only the socio-economic characteristics varied across groups. The coefficient effect (or unexplained component) estimates the difference that cannot be explained by dissimilarities in group composition and may be due to other behaviors specific to the group. To perform the decomposition, we used the “omega” option that estimates a two-fold decomposition using as references the coefficients estimated from a pooled model over both groups [42].

### Ethics

This paper is entirely and exclusively based on microdata from Spanish Vital Statistics. The information on births, obtained from bulletins originally registered in civil registers, is processed and offered by INE. All the data used in this study are available to the public. All the individual information contained in the microdata has been duly anonymized by INE. Hence, no ethical or governmental permissions were required for this study.

## Results

Considerable variations were observed in specific socio-demographic features between immigrant mothers of different origins residing in Spain. Descriptive results provided in Table 1 show these differences for the five immigrant categories considered in the analysis along with the Spanish native mothers. Immigrants from HICs are a highly distinctive migrant group, with remarkable differences from the other categories of immigrants, and many similarities with the native Spanish population (above all, in terms of birthweight, birth order, age of mothers and educational attainment of mothers and labor complications and cesarean deliveries). Mothers from HICs are on average older and have notably higher levels of SES than mothers from other origins, both in terms of educational attainment (the highest prevalence of tertiary education) and occupational class (the highest proportions of managers/professionals and white collar workers). In addition, mothers from high-income countries also had higher levels of intermarriage with Spanish partners (64% of the fathers of infants born to these HIC mothers are native Spanish), and almost 40% have Spanish citizenship, far more than mothers from NHI-EUR (1.5%), AFCs (6.8%), ASCs (5.2%), or LACs (31.8%). African mothers stand out in terms of parity (10% of AFC mothers registered their delivery as their fourth or higher child), formal partner relationships, and lower educational achievement and occupational status. LAC and NHI-EUR mothers are similar in terms of educational achievement and occupational status, although LAC mothers acquired Spanish citizenship much more often than NHI-EUR mothers, and the proportion of never married LAC mothers is considerably higher. Owing to a relatively lower educational attainment level (only higher than that of AFC mothers), workers in the service sector abound in the ASC families.

**Fig. 1:**
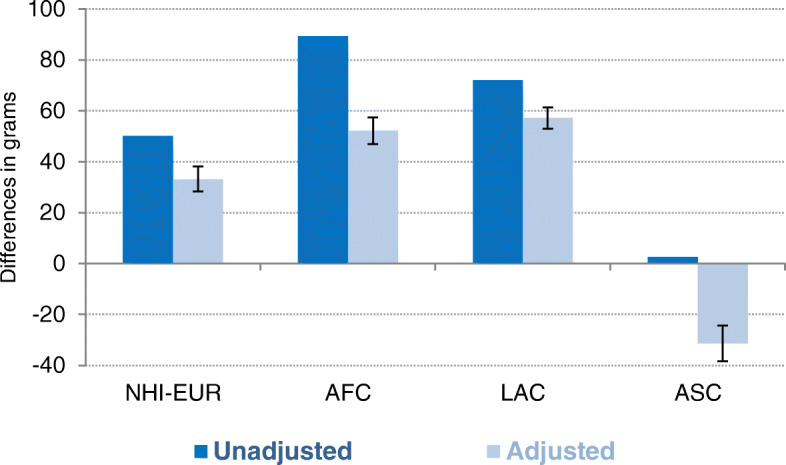
OLS regressions on mean birthweight differences in grams between singleton births in 2007–2017 to immigrant mothers from high-income countries (HICs) and other origins

Figure 1 shows the mean adjusted and unadjusted birthweight differences (in grams) between singleton births in 2007–2017 from HIC mothers and mothers from other origins. Globally, both non-adjusted and adjusted results confirmed the healthy immigrant paradox for immigrant populations in Spain. However, important variations were observed between immigrants from different origins. Infants born to HIC mothers have lower average birthweights than those born to NHI-EUR, AFC, and LAC mothers, but have similar birthweights to those born to ASC mothers. These differences vary in a range of 50.2 g for NHI-EUR mothers to 89.3 g for AFC mothers, or 1.5% to 2.7% of the total birthweight. On average, LAC mothers’ infants are 72.0 g heavier than those of HIC mothers, whereas ASC mother’s infants weigh only 2.6 g more than HIC mothers’ infants. After adjusting for the newborn’s sex, birth order, labor complications, caesarean delivery, age of mother, marital status of mother, education of mother, family class, citizenship of mother, age of father and migratory status of father, the differences persisted, although they were attenuated and followed a relatively similar pattern and equally ranked. Model estimates showed that NHI-EUR infants (33.2 g; 95% CI: 28.3–38.1, *P* < 0.01), AFC infants (52.2 g; 95% CI: 46.9–57.5, *P* < 0.01), and LAC infants (57.1 g; 95% CI: 52.9–61.3, *P* < 0.01) weigh more than HIC infants, whereas ASC infants weigh the least (31.4 g; 95% CI: 38.4–24.3, *P* < 0.01).

The regression model estimates (see Table 2) also confirmed the negative association of birthweight with female infants, unmarried mothers, and immigrant mothers having naturalized as Spanish citizens. In contrast, birthweight is positively associated with labor complications, caesarean deliveries, birth order, and two characteristics of the father: age and migrant status. Birthweight holds a curvilinear relationship with age (with heavier newborns born to 20–30-year-old mothers and smaller infants to mothers with extreme maternal ages) and education (smaller infants are more frequent among mothers with both less and more education). Finally, after controlling for educational achievement, family class did not appear to have any significant impact on the mean birthweight.

**Table 2:** OLS regression model on birthweight

		Coef.	Std. Err.	***P***-value	[95% Conf.	Interval]
Infant sex	Male	0				
	Female	− 124.129	1.243	0.000	− 126.565	−121.694
Birth order	1st	0				
	2nd - 3th	96.369	1.360	0.000	93.704	99.035
	4th+	139.752	3.191	0.000	133.497	146.007
Labor complications	No	0				
	Yes	31.561	2.141	0.000	27.366	35.757
Caesarean delivery	No	0				
	Yes	24.331	1.817	0.000	20.770	27.892
Age of mother	< 20	0				
	20–30	53.784	3.665	0.000	46.601	60.967
	31+	44.870	3.925	0.000	37.177	52.562
Marital status of mother	Married	0				
	Never married	−5.547	1.454	0.000	−8.397	−2.698
	Separated/Divorced	−8.937	3.167	0.005	−15.144	−2.730
	Widow	−36.169	14.020	0.010	−63.647	−8.691
Education of mother	Primary or less	0				
	Lower Secondary	16.919	1.811	0.000	13.369	20.468
	Upper Secondary	18.038	1.940	0.000	14.236	21.840
	University	10.156	2.409	0.000	5.436	14.877
Family class	Manager/Professional	0				
	White Collar	−3.126	3.021	0.301	−9.047	2.796
	Service Workers	5.753	2.976	0.053	−0.080	11.586
	Skilled Workers	9.467	3.339	0.005	2.922	16.012
	Unskilled Workers	6.414	3.175	0.043	0.192	12.637
	Inactive	−5.983	2.832	0.035	−11.533	−0.432
Citizenship of mother	Other	0				
	Spanish	−31.294	1.725	0.000	−34.676	−27.913
Age of father		0.442	0.104	0.000	0.239	0.645
Migratory status of father	Native	0				
	Migrant	22.839	1.558	0.000	19.786	25.893
Origin of Mother	HIC	0				
	NHI-EUR	33.208	2.500	0.000	28.307	38.108
	AFC	52.219	2.672	0.000	46.983	57.456
	LAC	57.147	2.143	0.000	52.947	61.347
	ASC	−31.365	3.606	0.000	−38.433	−24.298
Constant		3254.735	5.751	0.000	3243.463	3266.007

**Fig. 2:**
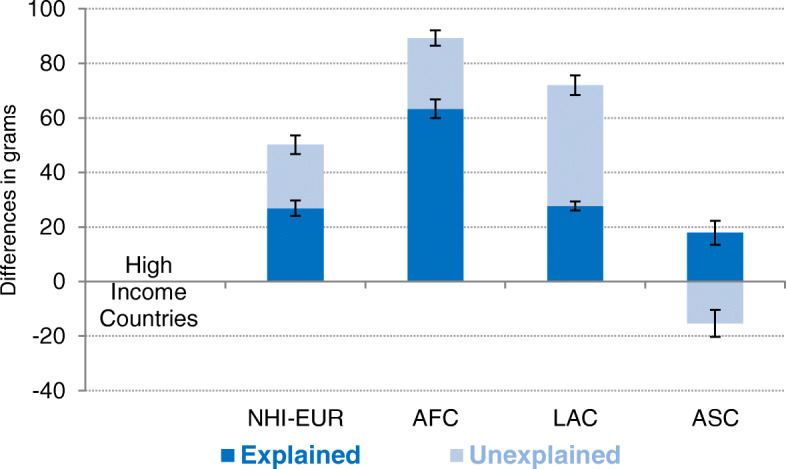
Oaxaca−Blinder linear regression-based decomposition of differences between immigrant mothers. Contribution of explained and unexplained components

These differences in birthweight across immigrant populations can be accounted for by the composition and/or the differential behaviors of the respective groups. To separate these two factors, we performed a regression-based decomposition of birthweight differences between HIC mothers and the other four categories of immigrant mothers. Figure 2 graphically reports the contribution (in grams) of both types of factors to the total difference in birthweight. This illustrates how much of these gaps can be attributable to differences in observable characteristics between groups (the explained portion or composition effects) and how much remain unexplained (the coefficient effect or the portion of differences that do not stem from observable distributions of characteristics). The explained portion indicates the mean increase in the birthweight of infants from immigrant mothers of a given origin if they had the same characteristics as HIC infants and mothers, respectively, but their own coefficients. The second (unexplained) term estimates the change in the birthweight of infants born to mothers of a given origin if the coefficients of HICs were to be applied to them, while maintaining their own characteristics. In other words, the explained portion measures the part of the difference between groups attributable to their composition (e.g., group differences in the predictors) while the unexplained term or coefficient effect refers to others non-compositional differences, included those due to unobserved predictors. Besides the mean birthweights for the four groups and differences between the four pairs, Table 3 reports in more detail the respective contributions of each covariable included in our models to the explained portion.

**Table 3:** Lineal decompositions of the differences in birthweight between HIC mothers and mothers of other origins

	NHI-EUR – HIC					AFC – HIC					LAC – HIC					ASC – HIC				
	Coef.	SD	P > z	[95%	CI]	Coef.	SD	P > z	[95%	CI]	Coef.	SD	P > z	[95%	CI]	Coef.	SD	P > z	[95%	CI]
**Overall**																				
HIC	3368.3	1.5	0.000	3365.4	3371.1	3407.4	1.3	0.000	3404.8	3410.1	3390.1	1.0	0.000	3388.2	3392.0	3320.7	2.9	0.000	3315.1	3326.3
Other origin	3318.1	1.8	0.000	3314.7	3321.6	3318.1	1.8	0.000	3314.7	3321.6	3318.1	1.8	0.000	3314.7	3321.6	3318.1	1.8	0.000	3314.7	3321.6
Difference	50.2	2.3	0.000	45.7	54.6	89.3	2.2	0.000	84.9	93.7	72.0	2.0	0.000	68.1	76.0	2.6	3.4	0.443	−4.0	9.2
Explained	26.9	1.4	0.000	24.0	29.7	63.3	1.7	0.000	60.0	66.7	27.7	0.8	0.000	26.1	29.3	17.9	2.3	0.000	13.5	22.4
Unexplained	23.3	1.8	0.000	19.8	26.8	26.0	1.4	0.000	23.1	28.8	44.3	1.9	0.000	40.7	47.9	−15.3	2.5	0.000	−20.3	−10.4
**Explained**																				
Infant sex	0.7	0.3	0.034	0.1	1.4	0.9	0.3	0.002	0.4	1.5	0.3	0.3	0.267	−0.2	0.8	1.2	0.4	0.005	0.4	2.1
Birth order	−3.8	0.2	0.000	−4.3	−3.3	18.8	0.5	0.000	17.7	19.8	5.7	0.3	0.000	5.2	6.2	10.0	0.5	0.000	8.9	11.0
Labor complications	−0.4	0.1	0.000	−0.6	−0.2	0.1	0.1	0.071	0.0	0.2	0.6	0.1	0.000	0.4	0.7	−0.5	0.1	0.000	−0.8	− 0.2
Caesarean delivery	−0.2	0.1	0.211	−0.5	0.1	−0.1	0.1	0.230	−0.2	0.1	0.9	0.1	0.000	0.7	1.1	0.0	0.1	0.895	−0.1	0.2
Age of mother	5.6	0.9	0.000	3.8	7.4	5.0	0.8	0.000	3.5	6.5	−0.1	0.4	0.765	−1.0	0.7	1.6	1.3	0.231	−1.0	4.2
Marital status of mother	0.8	0.1	0.000	0.6	1.0	6.5	0.8	0.000	5.0	8.1	0.2	0.1	0.212	−0.1	0.4	0.6	0.6	0.256	−0.5	1.7
Education of mother	−7.6	0.9	0.000	−9.4	−5.8	−1.0	1.8	0.560	−4.5	2.5	4.3	0.7	0.000	3.0	5.5	−8.7	2.0	0.000	−12.7	−4.7
Family class	−2.1	1.0	0.041	−4.1	−0.1	−1.6	1.6	0.325	−4.8	1.6	3.9	0.7	0.000	2.6	5.2	−9.9	1.6	0.000	−13.0	−6.8
Citizenship of mother	23.2	1.3	0.000	20.7	25.6	10.1	1.0	0.000	8.1	12.1	2.3	0.1	0.000	2.0	2.6	14.7	1.3	0.000	12.2	17.2
Age of father	−1.1	0.5	0.022	−2.0	−0.2	1.9	0.3	0.000	1.4	2.4	−0.4	0.3	0.081	−0.9	0.1	1.3	0.8	0.095	−0.2	2.9
Migratory status of father	11.7	1.1	0.000	9.6	13.8	22.8	1.7	0.000	19.5	26.1	10.2	0.5	0.000	9.1	11.2	7.5	2.1	0.000	3.4	11.6

In general, differences in birthweight obey both the composition (explained portion) and coefficient effects (unexplained portion), but vary in proportion depending on the region of origin. In a comparison between HIC and NHI-EUR infants, compositional differences were found to account for 26.9 (95% CI: 24.0–29.7, *P* < 0.01) out of the 50.2 g of overall difference in birthweight. In three categories, the NHI-EUR births are much more prevalent than HIC births, accounting for most of the difference: age of mother (5.6 g, 95% CI: 3.8–7.4, *P* < 0.01), non-Spanish citizenship (23.2 g, 95% CI: 20.7–25.6, *P* < 0.01), and immigrant father (11.7 g, 95% CI: 9.6–13.8, *P* < 0.01). Interestingly, differences in education reduced the gap between HIC and NHI-EUR birthweights by 7.6 g (95% CI: 9.4–5.8, *P* < 0.01) due to their concentration in the lower educational ranks. In the case of AFC infants, 63.3 (95% CI: 60.0–66.7, P < 0.01) of the 89.3 g of overall difference with HIC infants (70%) is accounted for by compositional effects. Among these, disparities in birth order (18.8 g, 95% CI: 17.7–19.8, *P* < 0.01), marital status of the mother (6.5 g, 95% CI: 5.0–8.1, *P* < 0.01), foreign citizenship of the mother (10.1 g, 95% CI: 8.1–12.1, *P* < 0.01), and immigrant father (22.8 g, 95% CI: 19.5–26.1, *P* < 0.01) account for more than half (65%) of the overall gap in birthweight between these groups. In turn, the contrast between HIC and LAC births revealed that the explained difference accounts for 27.7 g (95% CI: 26.1–29.3, *P* < 0.01), or 38.5%, of the overall difference, a proportion lower than the two previous comparisons. Birth order (5.7 g, 95% CI: 5.2–6.2, *P* < 0.01), educational status (4.3 g, 95% CI: 3.0–5.5, *P* < 0.01), and immigrant father (10.2 g, 95% CI: 9.1–11.2, *P* < 0.01) make the bulk of the difference. Finally, when birth outcomes of HIC and ASC mothers were compared, decomposition showed a different pattern, with compositional effects increasing the birthweight of Asian infants (+ 17.9 g, 95% CI: 13.5–22.4, *P* < 0.01), and unexplained factors decreasing it (− 15.3 g, 95% CI: − 20.3 to − 10.4, *P* < 0.01), with an overall balance of no significant difference. Among compositional factors that increase the birthweight of ASC newborns, birth order (10.0 g, 95% CI: 8.9–11.0, *P* < 0.01), foreign citizenship of the mother (14.7 g, 95% CI: 12.2–17.2, *P* < 0.01), and immigrant father (7.5 g, 95% CI: 3.4–11.6, *P* < 0.01) stand out as contributing factors.

## Discussion

This article contributes to the literature on disparities in perinatal health of immigrant populations by providing two main findings. First, our results on birthweight show strong population-based evidence of a large number of “healthy immigrants” residing in Spain. However, not every immigrant matches this description, as immigrant birth outcomes vary significantly depending on the origin of the immigrant mother.

Unadjusted differences in birthweight between HIC infants and ASC infants were negligible. When adjusted for several relevant covariates, the sign of difference became negative, and Asian infants were found to weigh less than HIC infants. These results concur with previous findings showing elevated rates of low birthweight and smaller infants among ASC mothers having migrated from low- and middle-income countries and living in high-income host countries [3, 4, 13, 24, 43, 44]. They are also consistent with more recent estimates reporting a higher prevalence of low birthweights in Southeast Asia, Western Asia, and particularly Southern Asia than in high-income regions of the world including Europe [45]. It is safe to say that, with respect to perinatal outcomes, ASC migrants in Spain should not be considered paradoxically healthy immigrants, but rather ones maintaining the low birthweight characteristics of their autochthonous populations.

In contrast, AFC, LAC and NHI-EUR mothers give birth to infants heavier than those from HIC mothers. By implication, these immigrant infants are also heavier than those born to Spanish native mothers. Moreover, AFC and LAC autochthonous populations are reported to have lower birthweights compared with European populations [45, 46]. Therefore, because immigrants from AFCs and LACs to Spain give birth to the heaviest children, they are very clear examples of the healthy immigrant paradox.

Our research confirms that the healthy immigrant paradox is not a universal phenomenon and depends strongly on immigrants’ origin. More precisely, mothers from high-income countries tend to be similar to Spanish native mothers but have worse birth outcomes than mothers from mid- and lower-income countries, except for Asian ones. This finding, which has already been observed in other countries [14, 47], implies that our insight into the healthy immigrants should move beyond the dichotomy native/foreign born and the simplistic consideration of all immigrant subpopulations as similar communities.

Second, our study contributes to a better understanding of the mechanisms behind the differential perinatal outcomes of immigrant mothers. Although several studies identified specific characteristics that might shape differences in birth weight among immigrant mothers residing in Spain [21, 29, 35, 48], they neglected the fact that those disparities might respond to differences in the composition of specific groups.

Our results show that compositional effects are especially salient among AFC newborns, as they account for 70% of the increase in birthweight compared with those in HIC newborns. Parity, the mother’s marital status and foreign citizenship, and ethnic intermarriage composition are the more important factors, with a very high prevalence of second and higher birth orders and married, foreign and homogamic mothers. For births to LAC mothers, composition effects account for almost 40% of the overall disparity with HIC mothers. In this case, compositional differences in high birth orders, intermediate levels of education, and ethnic endogamy are decisive. Immigrant mothers and infants from NIH-EUR countries show similar results to LAC mothers and infants in the portion of birthweight difference attributable to compositional effects, but the relevant characteristics for the explained portion of the gap are those related to citizenship of mothers and intermarriage, with high proportions of unnaturalized and homogamic mothers. Finally, among ASC infants, some compositional characteristics, such as birth order and a high prevalence of ethnic homogamy, tend to increase their birthweight and make up for the baseline disadvantages in birthweight.

## Conclusions

Our results imply that despite several unobserved characteristics that might play an important role in explaining differences in birthweight among immigrants in Spain, a significant portion of the advantages in birthweight between immigrant groups is attributable to the compositions of the groups. Composition effects have the benefit of being self-explanatory: differences simply arise from the dissimilar distributions of certain traits across groups. In other words, AFC, LAC, and NIH-EUR immigrants owe a significant part of the observable advantage in birthweight over HIC immigrants to their particular group compositions. Even ASC immigrant mothers offset part of their original basal disadvantage in birthweight thanks to their group composition. A significant part of the perinatal health advantages of certain immigrant groups is only a by-product of their group composition and does not correspond to medical, environmental, or behavioral factors.

### Limitations

Although important predictors of birthweight such as maternal age, birth order, multiple pregnancies and obstetric complications were controlled for in this study, other predictors were not. These uncontrolled predictors include chronic maternal conditions, infections and nutritional status, exposure to poor environmental conditions, and tobacco, alcohol, or drug consumption. A lack of control for these predictors is the main limitation to our study because these unmeasured predictors could influence the outcome, if it were possible to include them in the models. Nevertheless, part of the unobserved heterogeneity associated with these neglected predictors should have been absorbed by sociodemographic covariables, such as marital status, educational attainment, family social class, citizenship acquisition, and intermarriage, that were included in the models. Previous studies [49] have shown that even when maternal traits, such as height and weight, and other lifestyle and behavioral characteristics, such as smoking habits, are taken into account by models, and mothers with medical conditions (diabetes, preeclampsia) are excluded from the analysis, ethnic origin differences in birthweight still persist. In summary, we are confident that our results are robust in showing that dissimilarities in birthweight between immigrants of different origins vary from one origin to another. Not all immigrants can be considered as paradoxically healthy, and in cases where they are, an important part of the advantage should be attributable to their composition as a group.

## Data Availability

The datasets analyzed during the current study are available in the Spanish National Statistical Office (Instituto Nacional de Estadistica, INE) repository, https://www.ine.es/dyngs/INEbase/es/categoria.htm?c=Estadistica_P&cid=1254734710984

## Abbreviations

AFC: African countries
ASC: Asian countries
HIC: High Income Countries
INE: Instituto Nacional de Estadistica (Spanish National Statistical Office)
LAC: Latin American countries
NHI-EUR: Non-high-income European countries
OLS: Ordinary Least Square
SES: Socio-economic status

## Footnotes

1 Bootstrapping estimation with 50 repetitions produced similar results to those presented here.
